# Post-Ebola Syndrome, Sierra Leone

**DOI:** 10.3201/eid2204.151302

**Published:** 2016-04

**Authors:** Janet T. Scott, Foday R. Sesay, Thomas A. Massaquoi, Baimba R. Idriss, Foday Sahr, Malcolm G. Semple

**Affiliations:** University of Liverpool Institute of Translational Medicine & NIHR Health Protection Research Unit in Emerging and Zoonotic Infections, Liverpool, UK (J.T. Scott, M.G. Semple);; 34th Regimental Military Hospital, Freetown, Sierra Leone (F.R. Sesay, T.A. Massaquoi, B.R. Idriss, F. Sahr)

**Keywords:** Post-Ebola syndrome, sequelae, Ebola virus, viruses, Sierra Leone

## Abstract

Thousands of persons have survived Ebola virus disease. Almost all survivors describe symptoms that persist or develop after hospital discharge. A cross-sectional survey of the symptoms of all survivors from the Ebola treatment unit (ETU) at 34th Regimental Military Hospital, Freetown, Sierra Leone (MH34), was conducted after discharge at their initial follow-up appointment within 3 weeks after their second negative PCR result. From its opening on December 1, 2014, through March 31, 2015, the MH34 ETU treated 84 persons (8–70 years of age) with PCR-confirmed Ebola virus disease, of whom 44 survived. Survivors reported musculoskeletal pain (70%), headache (48%), and ocular problems (14%). Those who reported headache had had lower admission cycle threshold Ebola PCR than did those who did not (p<0.03). This complete survivor cohort from 1 ETU enables analysis of the proportion of symptoms of post-Ebola syndrome. The Ebola epidemic is waning, but the effects of the disease will remain.

## Abstract 2

Ebola survivors experience a range of medical conditions.

Thousands of persons have now survived Ebola virus disease (EVD). During efforts to control the current Ebola-Zaire outbreak, attention has focused on containing spread of infection and improving survival. In Sierra Leone, 4,051–5,115 persons are confirmed to have survived from among 8,704 confirmed cases and 3,589 confirmed deaths (*1*).

Survivors report a range of sequelae loosely described as post-Ebola syndrome. Follow-up clinics were not always planned as part of the emergency response. However, survivors from the Ebola treatment unit (ETU) at the 34th Regimental Military Hospital, (MH34), Wilberforce Barracks, Freetown, Sierra Leone, were all followed up in an outpatient clinic within 2 weeks after discharge. Although resources to care for survivors, including basic equipment (e.g., adequate stethoscopes), were scarce, each survivor was seen by a physician who made contemporaneous structured notes, which afforded an opportunity to document post-Ebola syndrome during these first weeks.

What proportion of Ebola survivors have sequelae is not clear. Little is known about post-Ebola syndrome or whether it is an entity distinct from an appropriate response to the traumatic events of EVD. Abdominal pain, vision loss, hearing loss, impotence, bleeding, psychological problems, and general weakness were listed qualitatively as symptoms of post-Ebola syndrome after the Ebola-Sudan outbreak in Uganda in 2000 (*2*). Arthralgia and ocular diseases were noted in 19 survivors (selected according to availability) who were followed up after the 1995 Ebola-Zaire outbreak in Kikwit (*3*,*4*); in the same outbreak, arthralgia, myalgia, abdominal pain, extreme fatigue, and anorexia were more common in Ebola survivors than in their household contacts (*5*). From the current outbreak, survivors reported arthralgia and anorexia (which in this context includes loss of appetite without weight loss) in a telephone-administered questionnaire in Guinea several months after discharge (*6*). Because none of these studies comprised an unselected cohort of survivors, interpretation of proportions was difficult. Other reports referred to anecdotes of pain, weakness, difficulty hearing, and mental disturbances (*7*,*8*). These observations suggest complaints that might be expected. Descriptions of the proportions of survivors needing care for the most common problems are needed to plan health care for the thousands of survivors. We report the symptoms described by all EVD survivors from 1 ETU in the initial weeks after discharge.

## Methods

The MH34 ETU can accommodate 30 persons with confirmed EVD plus 20 persons with suspected EVD; it also contains a doffing area. MH34 opened on December 1, 2014, with 115 staff, including 3 physicians and catered to patients who fell ill in western Freetown and surrounding areas. The ETU admitted 355 patients (84 PCR-positive patients) and discharged 44 survivors during December 2014–March 2015. The area for persons with confirmed EVD is a permanent building with several 1–4-bed rooms that have electric lighting and ceiling fans. Three hot meals per day are provided, generally rice with protein, such as fish or chicken; each meal is provided with 2 bags of water, and more water is freely available. Staff members of this small ETU are all permanent Sierra Leonean healthcare workers.

Patients were admitted to the confirmed Ebola ward when Ebola virus (EBOV) infection was confirmed by real-time PCR. For some patients, a cycle threshold (C_t_) result also was available. Although C_t_ results were not standardized between PCR platforms or between laboratories, a low C_t_ reflects a high viral load. Patients were staged on arrival to the ETU, as follows:

- Stage 1: influenza-like illness (i.e., fever, myalgia. lethargy, fatigue, headache, sore throat, conjunctival injection).
- Stage 2: multisystem features, including “wet” gastrointestinal symptoms (vomiting, diarrhea), neurologic symptoms (headaches, confusion), vascular symptoms and signs (capillary leak, respiratory distress, hypotension), rash.
- Stage 3: internal and external bleeding, multiorgan failure.

Patients were treated for Ebola with supportive care (*9*). Antimicrobial drugs were administered empirically, and artesunate, paracetamol, and 500 mL intravenous Ringer’s lactate were administered on arrival. Ongoing treatment included further boluses of intravenous fluid; antiemetic medication and proton pump inhibitors were administered in accordance with clinical need. Some patients participated in a compassionate use open nonrandomized study of a single unit of convalescent whole blood (CWB), results of which are pending.

Discharge criteria were as follows: 2 consecutive negative PCR results for Ebola virus on separate days; medical fitness, in the opinion of his/her physician; and adequate social provisions, including release of the house and household members from quarantine. During the convalescent period, many patients ate >1 serving of each meal, 3 time per day. Although they were not routinely weighed, most patients visibly gained weight.

On leaving the ETU, all survivors were issued a survivor’s certificate and invited to a follow-up appointment within 2 weeks after discharge. Some survivors were seen before this appointment because of clinical need.

Contact with survivors was maintained by mobile phone. Confirmation of identification has not proved problematic because the survivors and healthcare workers had come to know each other well. Appointments are made by mobile phone and unscheduled visits by patients to the hospital. All survivors attended their follow-up visits. Patients were examined by 1 of 3 experienced physicians.

A follow-up appointment was established as a standard of care in this ETU from the outset at the height of the epidemic. Handwritten clinical notes documented presenting complaints, symptoms, and signs. These notes were subsequently used to develop appropriate preprinted clinical documentation. Age, sex, presenting complaints, and history of transfusion with CWB were noted for each patient. Preexisting conditions were rare in this cohort of patients and not included in this data extraction. At that time, facilities and equipment for survivors were limited; for example, all stethoscopes had been incinerated; blood pressure cuffs, ophthalmoscopes, and specialist opinions were not available.

### Data Analysis

We determined 95% CIs and conducted hypothesis testing of binomial outcomes (binomial frequency test) continuous outcomes (Mann-Whitney U) and analyzed them using Stata version 9 (StataCorp LP, College Station, TX, USA). Graphics were produced by using Stata version 9 and R version 3.1.1 (R Foundation for Statistical Computing, Vienna, Austria).

## Results

### Demography

**Figure 1:**
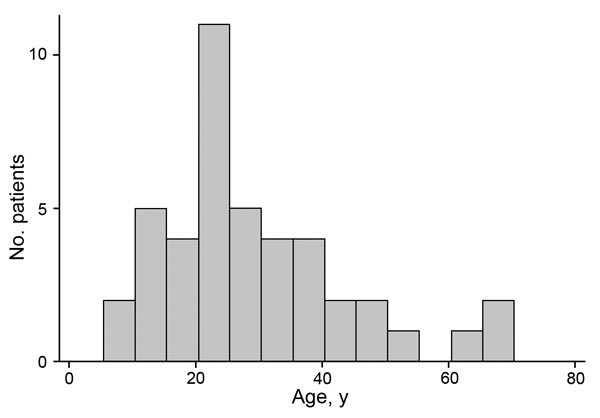
Age distribution of patients at Ebola survivors clinic at the 34th Regimental Military Hospital, Wilberforce Barracks, Freetown, Sierra Leone. Cycle threshold levels at hospital admission by age are shown in Table 1.

During December 1, 2014–March 30, 2015, the MH34 ETU treated 84 persons with PCR-confirmed EVD. Of these, 44 (52%) survived; 23 were female, and patient ages were 8–70 years (median 35 years; interquartile range [IQR] 20–37 years); age was not documented for 1 patient (Figure 1; Table 1).

**Table 1:** C_t_ results at hospital admission for 44 patients with post-Ebola syndrome, by sex, Sierra Leone*

Category	Patient sex		All patients
	M	F	
No. patients	21	22	44
C_t_ at admission			
Median	30	25	25
Range	10–52	8–70	8–70
IQR	22–37	20–34	20–37

*Age distribution of all patients is shown in Figure 1. C_t_, cycle threshold; IQR, interquartile range.

### Acute EVD Episode

Information about the acute EVD episode was available for 12 (27%) of the 44 survivors. Sex and ages of these 12 survivors did not differ significantly from those of the full set. For these 12 persons, median length of ETU stay was 15.5 days (range 9–17 days, IQR 13.5–16.6 days). For the 11 survivors for whom C_t_ results were available, median C_t_ at ETU admission (admission C_t_) was 28 (range 23–37, IQR 23–31). Two patients were admitted in clinical stage 1 and 9 in clinical stage 2. 

Twenty-three (52%) survivors received CWB. Ages of survivors receiving transfusions did not differ significantly from those of survivors who did not (p = 0.8). The frequencies of symptoms did not differ significantly between survivors who received CWB and those who did not (p = 0.5). Our study was not designed to assess efficacy or toxicity of CWB.

### Post-Ebola syndrome Complaints

At the time of data extraction, each survivor had attended at least 2 appointments. All survivors had >1 post-Ebola complaint (median 2, maximum 5). A total of 117 separate complaints were reported: 31 (70% [95% CI 55%–83%]) patients had musculoskeletal pain, 21 (48% [95% CI 32%–63%]) had headaches, and 6 (14% [95% CI 5%–27%]) had ocular problems.

**Figure 2:**
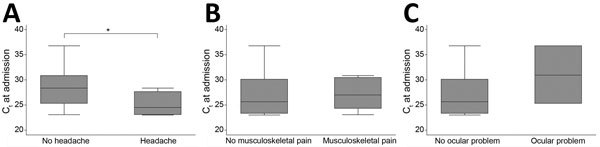
Comparison of the most common post-Ebola syndrome symptoms with admission C_t_ results, 34th Regimental Military Hospital, Wilberforce Barracks, Freetown, Sierra Leone. A) Headache, B) musculoskeletal pain, C) ocular problems. Specific C_t_ levels are shown in Table 2. *Indicates significant difference (p<0.03). C_t_, cycle threshold.

In their initial follow-up appointment, patients who reported headache had had admission C_t_ results that were significantly lower (correlating to a higher viral load) than those who did not subsequently report headache (with headache: n = 6, median C_t_ 24 [IQR 23–28]; without headache: n = 5, median C_t_ 31 [IQR 30–31]; p<0.03 by Mann-Whitney U test) (Table 2; Figure 2). There was no significant difference in admission C_t_ or clinical stage, or length of stay in the ETU for acute Ebola or clinical stage, between patients who had ocular problems or musculoskeletal pain and those who did not (Table 2; Figure 2). 

**Table 2:** C_t_ results at hospital admission for patients with post-Ebola syndrome who reported 1 of the 3 most common symptoms, Sierra Leone*

Category	Ocular problems	Musculoskeletal pain	Headache
Yes, no. patients	3	7	6
C_t_ at admission			
Median	31	25	24
IQR	25–37	23–30	23–28
No, no. patients	8	4	5
C_t_ at admission			
Median	27	29	31
IQR	23–29	25–34	30–31
p value†	0.2	0.5	**0.03**

*Box-and-whisker plots illustrating symptom appearances are shown in Figure 2. Some patients reported >1 symptom. Boldface indicates significance. C_t_, cycle threshold; IQR, interquartile range. †By Mann-Whitney U test.

One patient died after deteriorating respiratory symptoms and left-sided pleural effusion. He was a 25-year-old man in whom EVD was diagnosed on January 26; he received supportive care and 1 unit of CWB. His first negative PCR result was on February 8 and his confirmatory negative test on February 11; he was discharged home. At his 14-day follow-up visit, he had weight loss, cough, and dyspnea on exertion. At his second outpatient appointment, he was admitted to the general medical ward of MH34 on March 3 with left-sided pleural effusion. A pleural tap yielded only a small quantity of blood-stained fluid that was insufficient for analysis. He died on March 8, 2015, one month after his recovery from acute EVD. In adherence to safe-burial policy, a postmortem examination was not performed. His diagnosis remains unclear, but postviral effusion is possible, with tuberculosis pleural effusion a differential diagnosis.

#### Musculoskeletal Pain

Because in our experience and in the local context the distinction between myalgia and arthralgia can be physician-dependent, we merged these complaints. However, for the purpose of comparisons with other studies, we determined that 12 (27% [95% CI 15%–42%]) of the 44 survivors had arthralgia, 15 (34% [95% CI 20%–50%]) had myalgia, and 4 (9% [95% CI 3%–22%]) had both (Table 3). We found no significant differences between the proportion of male and female survivors, or between children (<18 years of age) and adults, who had musculoskeletal pain.

**Table 3:** Musculoskeletal symptoms described by 31 patients with post-Ebola syndrome, Sierra Leone*

Area of pain	Patient sex		Total
	M	F	
Joints			
Joint, unspecified	5	9	14
Knee, unspecified	2	0	2
Right knee joint	0	1	1
Shoulder joint	1	1	2
Body			
Generalized body	4	4	8
Upper back	1	3	4
Musculoskeletal, unspecified	2	0	2
Left thigh	1	1	2
Lower limb	0	1	1
Right thigh	1	0	1
Gluteal muscle	1	0	1

*Values are no. patients. Some survivors reported >1 area of pain. The proportion of male and female survivors with musculoskeletal pain did not differ significantly (χ^2^, p = 0.7).

Patients described musculoskeletal pain variously as problems with walking or moving or pain specific to 1 area (such as knees, thighs, or back) or generalized musculoskeletal pain. (21%–52%). Most often, patients characterized their musculoskeletal pain as a general pain rather than pain in a specific joint or area, as reflected in the recorded symptoms. Unspecified joint pain accounted for 14 of the 19 times joint pain was recorded (73% [95% CI 49%–90%]) and generalized body pain for 8 of the 19 times body pain was recorded (42% [95% CI 20%–67%]). Some patients recorded >1 symptom.

Examination indicated no joint inflammation or effusion, such as might be expected in a reactive condition, and patients retained full range of movement. Functional disability ranged from mild to moderate. For example, 1 man in his 20s continues to play football but now takes acetaminophen to facilitate this activity. A woman in her 40s requires assistance to step into a bath and cannot continue normal household work; she walked unaided into the clinic but needed assistance to step up into the clinic room and to sit and stand. Most of her musculoskeletal symptoms are relieved by simple analgesics.

#### Headache

Of the 21 (48% [95% CI 32%–63%]) survivors who reported having headache, 2 (10% [95% CI 1%–30%]) were girls 8 and 11 years of age. The proportion of male and female survivors reporting headaches did not differ significantly (p = 1 by χ^2^ test). Headache was generally described as affecting the full head, with no diurnal pattern and being constant. Ocular symptoms might coincide, but no visual phenomena, such as might be found in migraines, were reported.

##### Ocular Symptoms

Among the 6 (14% [95% CI 5%–27%]) survivors who reported ocular problems, symptoms were eye pain, clear discharge, red eyes, and blurred vision (Table 4). These symptoms appeared within 2 weeks after discharge and were not present at or before ETU discharge. Eye discharge was treated with topical chloramphenicol. Ophthalmology services for survivors are currently under development.

**Table 4:** Ocular symptoms described by 6 patients with post-Ebola syndrome, Sierra Leone

Patient age, y/sex	Symptom
8/F	Eye pain
14/F	Clear eye discharge
20/F	Clear eye discharge
28/F	Red eyes and blurred vision on the left
29/F	Red eyes
46/M	Blurred vision

#### Combinations of Musculoskeletal Pain, Headache, and Ocular Problems

**Figure 3:**
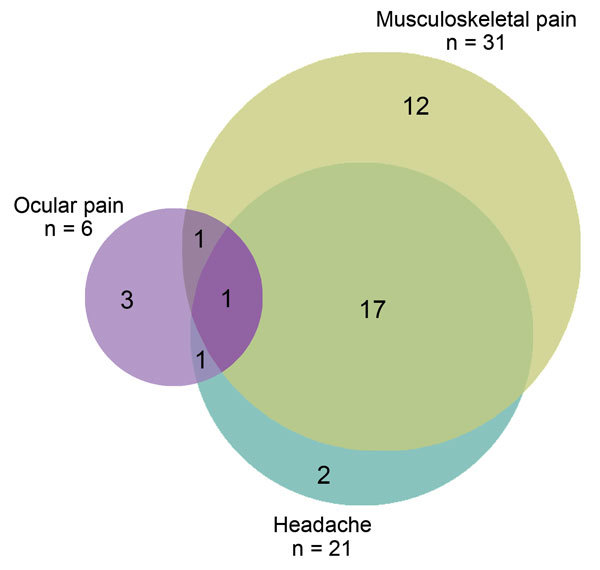
Scale Venn diagram illustrating the overlap between the 3 main symptom groups among persons with post-Ebola syndrome seen at the Ebola survivors clinic at the 34th Regimental Military Hospital, Wilberforce Barracks, Freetown, Sierra Leone. Seven patients did not have any of the 3 main symptom groups.

Musculoskeletal pain and headache overlapped substantially. Eighteen (58% [95% CI 40%–75%]) of the 31 survivors with musculoskeletal pain reported headache, and 18 (86% [95% CI 64%–97%]) of the 21 survivors with headache reported musculoskeletal pain. Two (6% [95% CI 1%–21%]) survivors with musculoskeletal pain reported ocular problems, and 2 (33% [95% CI 4%–78%]) with ocular problems reported musculoskeletal pain. Two (6 % [95% CI 1%–30%]) survivors with headache reported ocular problems. One survivor had all 3 complaints (i.e., 3% [95% CI 1%–17%] of survivors with musculoskeletal pain; 5% [95% CI 0%–24%] of those with headache, and 17% [95% CI 0%–64%] with of those with ocular problems) (Figure 3).

#### Other Symptoms

Twenty-six (59% [95% CI 43%–74%]) of the 44 survivors reported other symptoms. Five (11% [95% CI 4%–25%]) reported cough; 4 (9% [95% CI 3%–22%]) each reported abdominal pain, chest pain, or itching; 3 (7% [95% CI 1%–19%]) each reported insomnia, fever, or loss of appetite; 2 (5% [95% CI 1%–15%]) each reported labored speech, epigastric pain, or rash; and 1 (2% [95% CI 0%–12%]) reported weight loss, hiccups, increased appetite, chest pain, sneezing, diarrhea, vomiting, left sided weakness with facial nerve palsy, breathlessness, rash, dry flaky skin, earache, fever blister/cold sore, left scrotal swelling, nasal congestion, and tremors (Table 5).

**Table 5:** Post-Ebola complaints other than headache, musculoskeletal pain, or ocular problems among 44 survivors, Sierra Leone

Complaint	No. (%; 95% CI, %)
Cough	5 (11; 4–25)
Abdominal pain	4 (9; 3–22)
Chest pain	4 (9; 3–22)
Itching	4 (9; 3–22)
Insomnia	3 (7; 1–19)
Fever	3 (7; 1–19)
Loss of appetite	3 (7; 1–19)
Labored speech	2 (5; 1–15)
Epigastric pain	2 (5; 1–15)
Rash	2 (5; 1–5)
Other*	1 (2; 0–12)

*Weight loss, hiccups, increased appetite, chest pain, sneezing, diarrhea, vomiting, left sided weakness with facial nerve palsy, breathlessness, rash, dry flaky skin, earache, fever blister/cold sore, left scrotal swelling, nasal congestion, tremors.

## Discussion

We documented symptoms of EVD survivors in the initial 3 weeks after negative Ebola virus PCR results and 2 weeks after ETU discharge. The dominant clinical features of this survivor cohort were musculoskeletal pain, headache, and ocular problems. Symptoms did not differ by survivor sex or age. Symptoms did not appear to be affected by use of CWB to manage acute EVD; however, this finding should be interpreted with caution because this report is not a prospective study and not designed to consider the effect of CWB on post-Ebola syndrome. Whether this collection of signs and symptoms after acute EVD constitutes a separate syndrome might be semantic. Because experience of survivors in the weeks after EVD, although varied, has common features, we propose that the term post-Ebola syndrome is useful to describe these phenomena.

Our findings are consistent with some aspects of previous reports (*2*,*5*) but vary from others. For example, the prevalence of extreme fatigue and anorexia reported in Kikwit and Guinea (*5*,*6*) was not dominant in the cohort reported here. This finding might be due to the period of inpatient convalescence of survivors at MH34 with substantial nutritional support.

We hypothesize that the pathogenesis of pain, particularly muscle pain, is a sequelae of widespread myositis or rhabdomyolysis during acute EVD. This hypothesis would be consistent with laboratory data reporting raised transaminases and disseminated intravascular coagulation from a previous outbreak of Ebola (*10*) in Sudan. Future research would benefit from a comparison of a survivor cohort with a matched group who had not had Ebola and, if this pain is more common in Ebola survivors (as was found in Kikwit [*5*]), further elucidation of its etiology would be useful in determining treatment strategies.

Post-Ebola syndrome includes, but is not restricted to, musculoskeletal pain, headache, and ocular problems. Because some complications occur weeks or months after acute onset of EVD, some symptoms might be underestimated in this cohort (*2*,*5*). Since these data were extracted, clinical facilities and documentation has improved, so future information is likely to be more detailed in terms of specific diagnosis, and scope, particularly in regard to psychosocial health and ophthalmology. Previous outbreaks have reported psychosocial problems (*2*), although they are not included in all reports (*5*). Psychosocial problems also were evident in the survivors in our study but not captured in the documentation. Improved collaboration with MH34’s mental health team should improve both the care and documentation. Anecdotal evidence from the survivors’ clinic suggests that more subtle neurologic problems, such as specific nerve palsies, might feature more heavily in a follow-up study.

Survivors who reported headache had had lower C_t_ results than did those who did not. Although patients with higher initial viral loads might be more likely to have central nervous system involvement, and then have a higher probability of headache as a post-Ebola sequelae; C_t_ values are not standardized among platforms or laboratories. This intertest variability, together with the small sample sizes in this data extraction, suggests any association should be interpreted with caution. We propose that this association warrants further investigation. Headaches could also represent ongoing tension headaches or might result from underlying undiagnosed changes in vision.

We would expect the criteria and definition of post-Ebola syndrome to continue to develop and that the survivors will continue to face fresh challenges. During the height of the Ebola epidemic, when these consultations took place, resources and equipment for assessing survivors were limited. Our survey documents symptoms only in the first 3 weeks after ETU discharge. Subsequent follow-up might be more detailed and benefit from increased resources, and symptoms continue to develop with time. Indeed, Ebola virus can cross the blood–brain barrier during the acute illness (*11*) and persists in some compartments for several months (*12*). Areas for development include comparison of symptoms to community controls, psychosocial problems, causes of ocular problems and musculoskeletal pain, and longitudinal description of the clinical picture.

Because musculoskeletal pain is a common complaint in the general population in Sierra Leone, a community-controlled comparison is needed. In survivors of the Kikwit Ebola-Zaire outbreak in 1995, Rowe et al. reported that their key features—arthralgia, myalgia, abdominal pain, fatigue, and anorexia—were more common in survivors than in household contacts, whereas fever, headache, diarrhea, dyspnea, hiccups, and hemorrhage were the same in both groups (*5*). A topic for future research is the longitudinal course of recovery. Wendo et al. (*2*) reported that 1 year after the Ebola-Zaire outbreak in Uganda, 25% of patients were still reporting to clinic. Therefore, we can expect some survivors to have long-term clinical needs. The epidemic is waning but the effects of the disease it caused will remain.
